# Removal of the Willoughby Medical College to Columbus

**Published:** 1847-06

**Authors:** 


*Removal of the Willoughby Medical College to Columbus*—The Medical Institution at Willoughby, Ohio, has been transferred to Columbus, the trustees acting in accordance with an act of the Legislature to that effect. The corps of Professors has been re-organized, the following constituting the newly appointed Faculty:—

Anatomy and Physiology, J. P. Judkins, M. D., of Cincinnati.

Surgery, R. S. Howard, M. D., of Columbus.

Chemistry, Rev, F. Merrick, of Delaware.

Obstetrics and Diseases of Women and Children, H. H. Childs, M. D., of Massachusetts.

Pathology and Practice, J. Butterfield, M. D., of Columbus.

Materia Medica and Therapeutics, T. R. Spencer, M. D., of New-York.

Medical Jurisprudence and Insanity, S. M. Smith, M. D., of Columbus.

